# Ectopic breast fibroadenoma of the vulva: a case report

**DOI:** 10.3389/fonc.2025.1602250

**Published:** 2025-09-17

**Authors:** Yixiao He, Gang Xie, Yuzhu Ji, Yu Shi, Yushuang He, Xue Lei

**Affiliations:** Mianyang Central Hospital, School of Medicine, University of Electronic Science and Technology of China, Mianyang, China

**Keywords:** ectopic, breast fibroadenoma, vulva, diagnosis, treatment

## Abstract

**Background:**

Vulvar ectopic breast fibroadenoma (EBF) is an exceptionally rare benign neoplasm that lacks distinctive clinical or radiological features rendering pre-operative differentiation from other vulvar masses challenging. Definitive diagnosis requires histopathological confirmation. Two non-exclusive histogenetic hypotheses have been proposed: (1) derivation from ectopic breast tissue along the embryonic “milk line,” (2) origin from hormonally responsive anogenital mammary-like glands with latent potential for benign or malignant transformation.

**Case presentation:**

A 41-year-old woman presented with an incidentally discovered, slowly enlarging, painless right vulvar mass. Ultrasonography revealed a well-circumscribed hypoechoic nodule. Complete surgical excision was performed, and histopathological evaluation—including immunohistochemistry for estrogen receptor, progesterone receptor, GATA3, and p63—confirmed ectopic breast fibroadenoma. No recurrence was detected at 3-month follow-up.

**Conclusion:**

Despite its rarity, vulvar EBF must be considered in the differential diagnosis of vulvar masses in reproductive-age women. En-bloc excision is curative; however, long-term surveillance is warranted to monitor for hormone-driven recurrence or malignant evolution.

## Introduction

Ectopic breast tissue (EBT) is defined as breast parenchyma situated outside the orthotopic mammary ridge. It is reported in 2–6% of the general population, affects both sexes, and appears to be most prevalent in Japanese women (1). The axilla is the most common site of presentation (2), vulvar involvement is exceptional. Analogous to eutopic breast tissue, EBT can give rise to the full spectrum of benign and neoplastic lesions, often creating diagnostic confusion with primary vulvar tumors. Fibroadenoma arising within vulvar EBT is exceedingly rare (3, 4). We herein describe a 41-year-old woman who presented with a progressively enlarging vulvar mass. Pre-operative ultrasound revealed a well-circumscribed hypoechoic nodule in the subcutaneous tissue of the right labium majus. Complete surgical excision was performed in our Gynecology Department, and histopathologic examination confirmed ectopic breast fibroadenoma. This report, together with a focused review of the literature, outlines the clinical, radiologic, and pathologic features of vulvar ectopic breast fibroadenoma and discusses diagnostic strategies and prognosis.

## Clinical data

A 41-year-old woman first noted an asymptomatic vulvar mass 10 months before presentation. Because the lesion was neither pruritic nor painful, she did not seek medical attention. Seven days prior to admission she perceived rapid enlargement and consulted our institution. On gynecological examination, a solitary, firm, well-circumscribed, mobile nodule measuring approximately 2cm was palpable in the subcutaneous tissue of the right labium majus, the overlying skin was intact and non-tender. Pelvic examination revealed an anteverted, normal-sized, mobile uterus with a smooth surface and no adnexal masses or tenderness. Her gynecologic history was unremarkable, apart from one cesarean section in 2009. She currently uses barrier contraception with condoms. Bilateral breasts were symmetric, without palpable masses or accessory tissue. The patient denied cyclical variation in nodule size or associated mastalgia and reported no family history of breast or vulvar neoplasms. Based on these findings, the pre-operative differential diagnosis favored benign entities such as epidermal inclusion cyst, hemangioma, lipoma, or cutaneous adnexal tumor.

## Auxiliary examination

**Figure 1:**
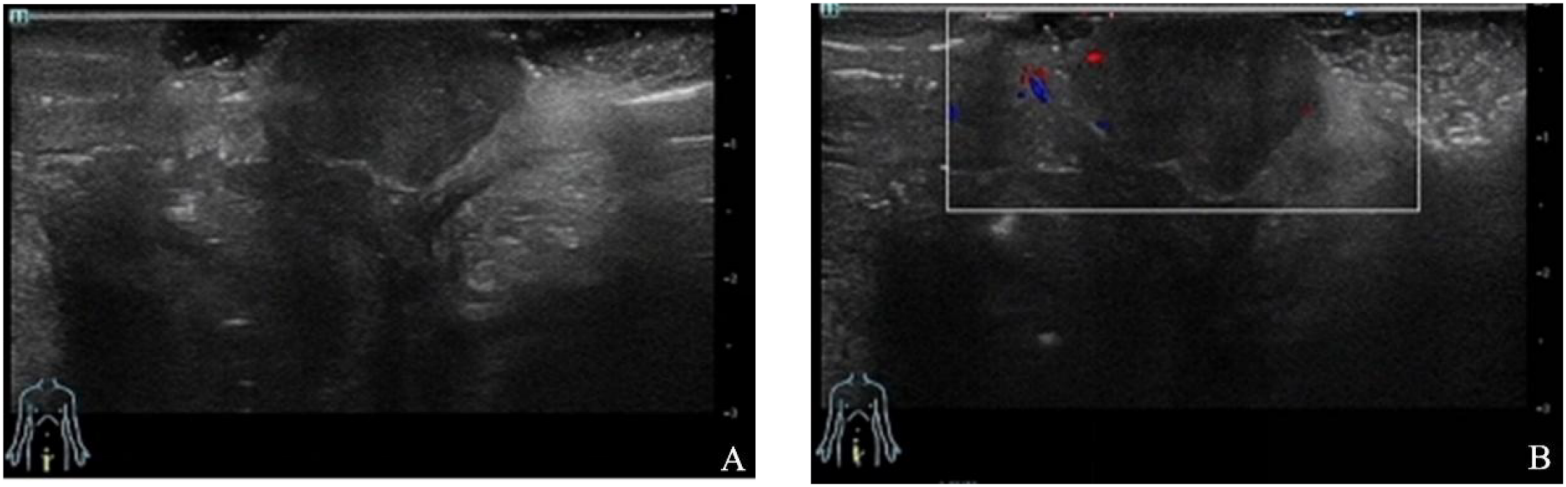
Ultrasound revealed a hypoechoic nodule with clear boundaries in the subcutaneous tissue of the right vulva.

Ultrasound revealed a hypoechoic nodule measuring approximately 2.0 * 1.3 * 1.2 cm with clear boundaries in the subcutaneous tissue of the right vulva, suggesting a subcutaneous hypoechoic nodule in the right vulva (**Figure 1**).

## Intraoperative findings

**Figure 2:**
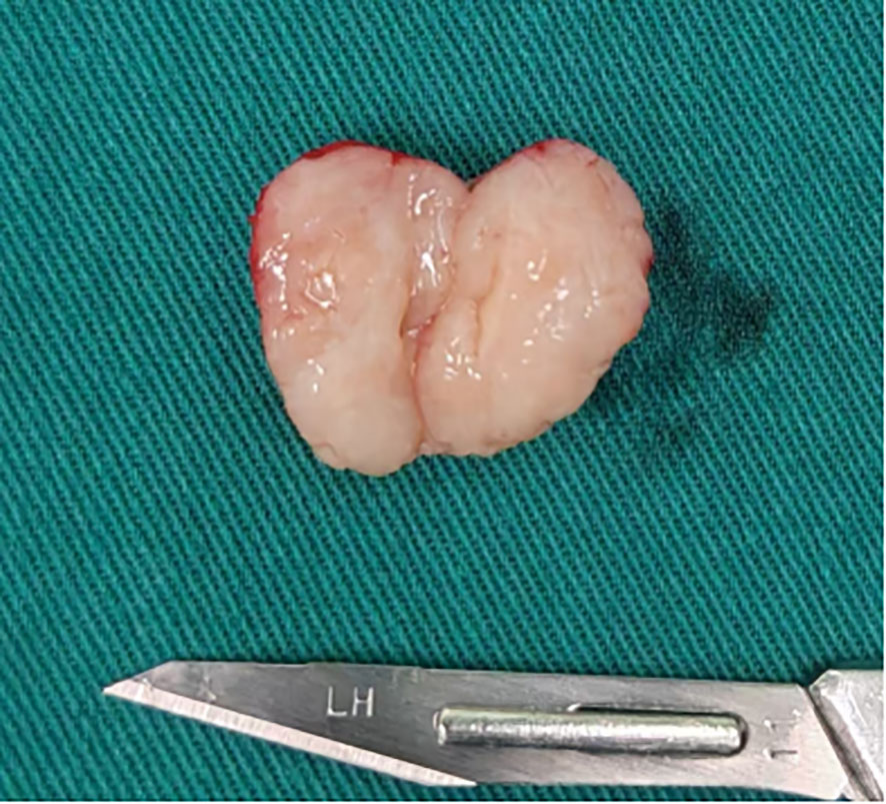
The mass was an oval shape with a complete thin fibrous capsule and a grayish-white solid tissue surface that was slightly soft.

Under sterile conditions, the lesion was excised en bloc via high-frequency electrosurgery. A well-encapsulated, firm nodule measuring 2.0 cm × 1.5 cm was identified immediately inferior to the perineal commissure at the lower margin of the right labium majus. The overlying capsule was thin and intact. Sectioning disclosed an ovoid, grayish-white, solid cut surface with a homogeneous, slightly soft consistency and no gross evidence of hemorrhage or necrosis (**Figure 2**).

## Pathological examination

**Figure 3:**
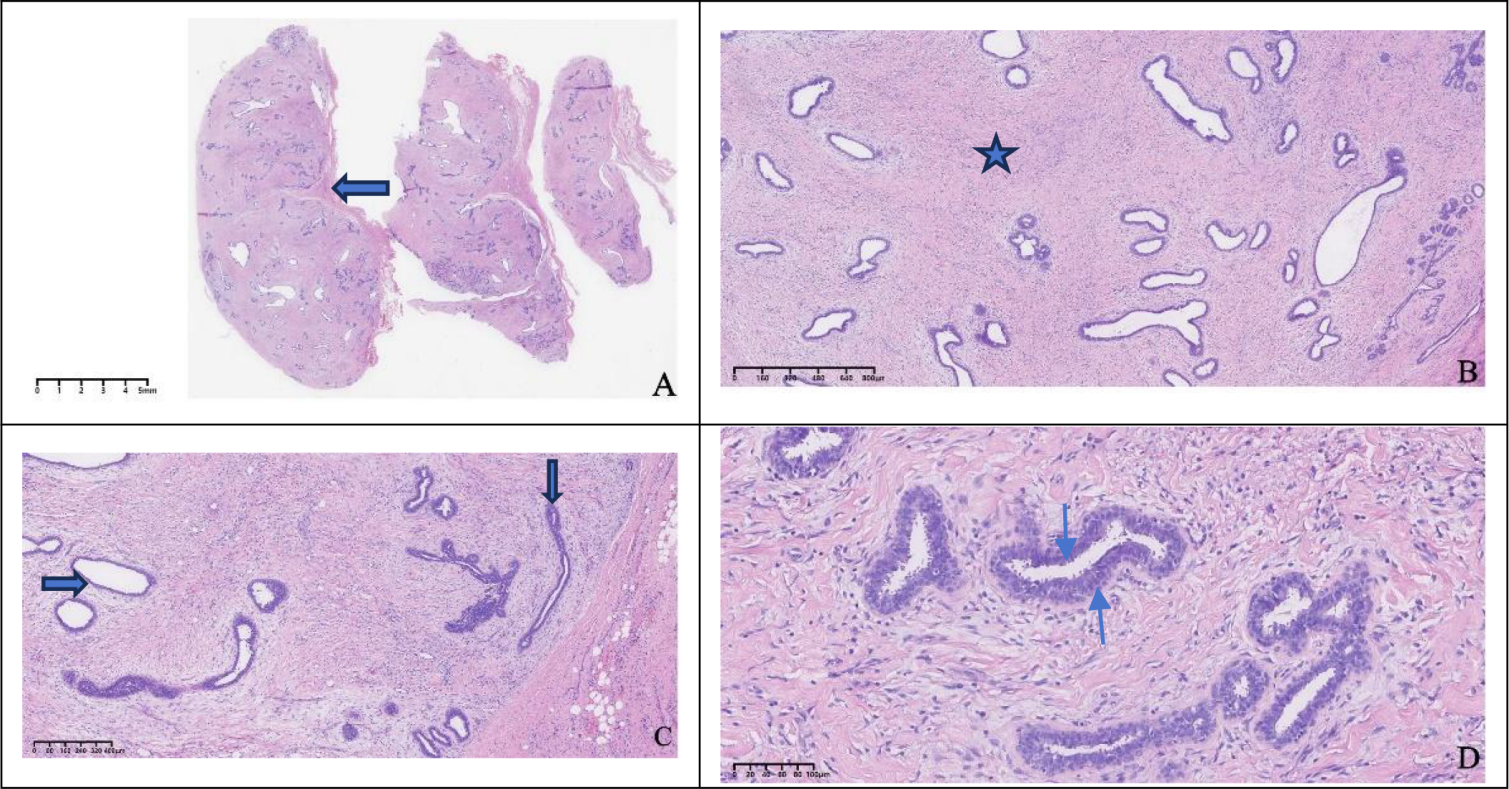
Histological morphology. The tumor surface was smooth with a thin fibrous capsule (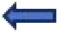), 2X **(A)**, mildly cellular stroma was observed within the tumor (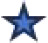), with numerous proliferative glandular structures, some lumina were dilated (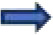), while in some areas the lumina were compressed and closed (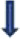), 4X,10X **(B, C)**, the glands were composed of an outer myoepithelial layer (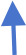) and an inner glandular epithelial layer (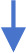), 20X **(D)**.

**Figure 4:**
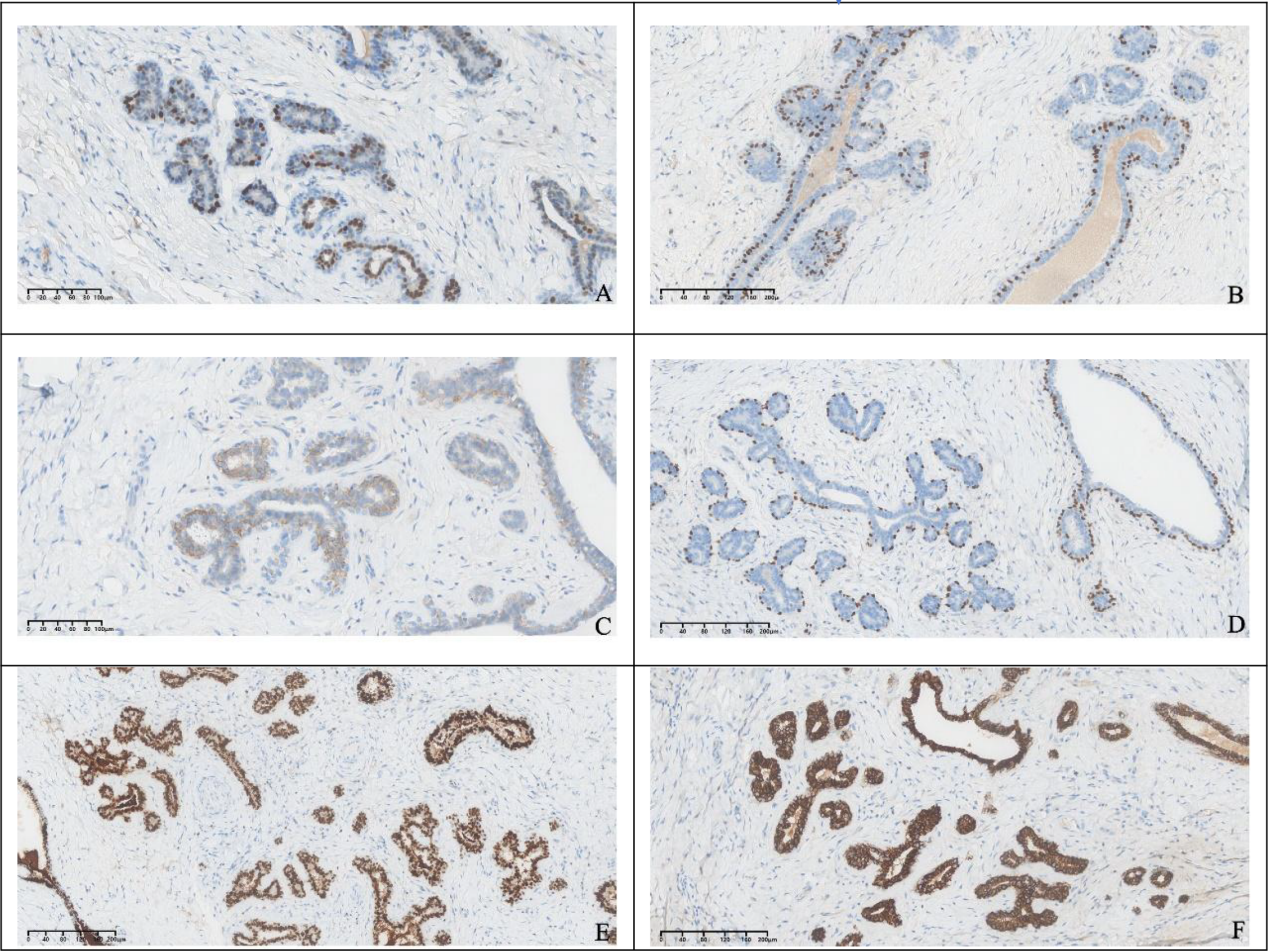
Immunohistochemical staining showed that the glandular epithelium was positive for ER, PR, HER2 **(A–C)**, the outer myoepithelial layer was positive for P63 **(D)**, GATA3 and E-Cadherin demonstrated positivity for both glandular epithelium and myoepithelial **(E, F)**.

The excised specimen consisted of a well-circumscribed, ovoid mass measuring 2.6×2.2×1.0 cm. The external surface was smooth and enveloped by a delicate, intact fibrous capsule. Cut section revealed homogeneous, grayish-white tissue lacking hemorrhage or necrosis. Microscopically, the lesion was circumscribed by a thin fibrous pseudocapsule and composed of mildly cellular fibrous stroma harboring proliferative, variably configured glandular structures. The glands exhibited a bilayered architecture comprising an inner luminal epithelial layer and an outer myoepithelial layer, recapitulating native breast tissue. Some ducts were mildly dilated; others were compressed or slit-like (**Figure 3**). Immunohistochemical staining showed that the glandular epithelium was positive for ER, PR, HER2, the outer myoepithelial layer was positive for P63, GATA3 and E-Cadherin demonstrated positivity for both glandular epithelium and myoepithelial. Therefore, based on the histological appearance and immunohistochemical staining results, we diagnosed it as an ectopic breast fibroadenoma (**Figure 4**).

## Follow-up

Three months post-operatively, clinical and high-resolution ultrasound re-evaluation demonstrated complete excision with no evidence of residual or recurrent disease within the vulva.

## Discussion

Vulvar ectopic breast fibroadenoma is an exceptionally rare entity whose pathogenesis remains incompletely understood. Two principal hypotheses have been proposed. The classic “milk-line” theory postulates that the lesion derives from residual embryonic mammary ridges that normally regress except for the bilateral pectoral primordia; incomplete involution along the caudal extension of this line could, albeit rarely, give rise to ectopic breast tissue in the vulva (5). An alternative model, advanced by Van der Putte, invokes the presence of anogenital mammary-like glands—specialized genital-derivative structures located in the interlabial sulcus and perianal region that are capable of estrogen- and progesterone-responsive secretory activity (6).These glands retain the potential for both benign and malignant transformation. Supporting this concept, phyllodes tumors exhibiting breast-like differentiation have been documented in extra-milk-line sites such as the prostate (7), implying that bona fide mammary epithelium is not an obligate prerequisite for such neoplasms. Collectively, these data suggest that vulvar breast fibroadenomas are more likely to originate from anogenital mammary-like glands rather than from aberrant migration along the embryonic milk line.

Aberrant breast tissue (ABT) is identified in approximately 1–3% of the general population, yet fibroadenoma developing within ABT is distinctly rare. More than 90% of reported cases occur in the axilla; the vulva constitutes the second most common site. Isolated lesions have also been described in the abdominal cavity, pelvis, chest wall, suprapubic region, periumbilical subcutis, and upper extremity (8–12). Within the limited vulvar cohort reported to date, patients are almost exclusively women of reproductive age, with a peak incidence between 30 and 50 years; presentation before the age of 20 or after 65 is exceptional. Vulvar lesions have additionally been documented during pregnancy and lactation (13). Owing to the rarity of the condition, current understanding derives solely from sporadic case reports and small case series; robust epidemiologic data are lacking. Consequently, the absence of pathognomonic clinical or imaging features renders pre-operative diagnosis challenging.

Patients usually present with a solitary, painless vulvar nodule that is well-circumscribed, mobile, and firm to rubbery on palpation. Growth is characteristically indolent, with progressive enlargement documented over months to years. The lesion is almost invariably unilateral, and reported diameters range from 1–2 cm to ≥ 5 cm. The labium majus is the most common site; involvement of the labium minus, periclitoral region, or perineal body is exceptional (14).

Although the lesion comprises ectopic mammary parenchyma, only a minority of women report mild premenstrual tenderness or subtle fluctuation in size; this cyclical change is inconsistent and markedly less pronounced than that typical of orthotopic fibroadenomas, and most patients remain asymptomatic. When progressive enlargement occurs in this anatomically intimate region, pain and mechanical irritation frequently ensue, prompting avoidance of sexual intercourse. Persistent discomfort may subsequently evolve into hypoactive sexual desire and impaired arousal. Alteration of vulvar contour often provokes embarrassment or body-image dissatisfaction, while friction from sitting, cycling, or tight clothing further compromises occupational, athletic, and social functioning. The cumulative impact of these factors ultimately compels presentation for medical evaluation.

At presentation, the rarity of the condition and its nonspecific clinical picture—together with imaging findings that merely suggest a benign neoplasm of indeterminate histogenesis—lead clinicians to favour more common entities such as epidermal inclusion cyst, lipoma, leiomyoma, or skin-appendage tumours. Consequently, ectopic mammary fibroadenoma is often overlooked. Even core-needle biopsy may only disclose a benign fibroepithelial lesion without identifying its aberrant mammary origin. The Kajawa–Ancillary Breast Tissue Classification System integrates macroscopic and microscopic characteristics to standardize the categorization of ABT and associated pathologies (**Table 1**). This framework is particularly valuable for multidisciplinary decision-making among breast surgeons, plastic surgeons and pathologists. Types I–VII are managed according to symptomatology (pain or cosmetic concerns), whereas Type VIII mandates complete surgical excision with mandatory histopathologic evaluation to exclude malignancy.

**Table 1:** Kajawa–ancillary breast tissue classification system.

Type	Designation	Characteristic description
Type I	A fully developed supernumerary breast.	characterized by the presence of a nipple, areola, and fully differentiated glandular tissue, thereby mirroring the macroscopic configuration of a native breast
Type II	glandular tissue and areola without an associated nipple	Presence of an areola and fully differentiated glandular parenchyma, with complete absence of a nipple structure.
Type III	Glandular tissue and nipple, in the absence of an areola.	Presence of a nipple and fully differentiated glandular tissue with concomitant absence of the areola.
Type IV	Isolated fully differentiated glandular tissue	Consisting solely of mammary glandular parenchyma without nipple or areola— the most prevalent variant that is frequently misinterpreted as lipoma or lymphadenopathy.
Type V	An areola with superimposed terminal hair follicles, lacking underlying glandular parenchyma.	Presence of an isolated areola, occasionally adorned by terminal hair follicles, yet devoid of functional glandular parenchyma—clinically masquerading as a pigmented nevus.
Type VI	Nipple only	Isolated nipple structure, devoid of both areola and underlying glandular parenchyma—frequently mistaken for an acrochordon or dermatofibroma.
Type VII	Hypertrichotic accessory breast presenting with hirsute areola lacking both nipple and glandular tissue.	Periareolar hypertrichosis, occasionally accompanied by involuted glandular remnants; clinical distinction from sebaceous cysts is mandatory.
Type VIII	Accessory breast tissue exhibiting pathological alterations.	Any of the aforementioned subtypes complicated by cyst formation, fibroadenoma, inflammatory change, or malignant transformation—mandating histopathologic verification.

Consequently, complete surgical excision remains the therapeutic gold standard for vulvar ectopic mammary fibroadenoma. Although recurrence is rare, documented cases implicate two principal mechanisms: (i) hormonal activation of residual mammary-like tissue during pregnancy, lactation, puberty, or ovarian hyper-stimulation, and (ii) persistence of microscopic disease at the resection margin. In addition, vulvar ectopic mammary lesions have been reported in association with congenital anomalies of the urinary tract, including hydronephrosis, polycystic kidneys, ureteric strictures, and adrenal hyperplasia (3). Therefore, dedicated renal imaging should be considered once the diagnosis is confirmed.

## Conclusion

Vulvar ectopic mammary fibroadenoma is an exceptionally rare benign neoplasm whose nonspecific clinical and imaging characteristics render accurate pre-operative diagnosis challenging. This case highlights the imperative of including mammary-type lesions in the differential diagnosis of any vulvar mass, particularly in women of reproductive age. En-bloc surgical excision provides excellent prognosis with minimal recurrence; however, long-term surveillance is warranted to monitor for hormonal stimulation and rare malignant transformation. Future studies should clarify the molecular pathogenesis and establish evidence-based management algorithms for this uncommon entity.

## Data Availability

The original contributions presented in the study are included in the article/supplementary material. Further inquiries can be directed to the corresponding authors.

## References

[1] Baradwan S Wadi KA . Unilateral ectopic breast tissue on vulva in postpartum woman: A case report. Med (Baltimore). (2018) 97:e9887. doi: 10.1097/md.0000000000009887, PMID: 29419702 PMC5944668

[2] Conde DM Kashimoto E Torresan RZ Alvarenga M . Pseudomamma on the foot: an unusual presentation of supernumerary breast tissue. Dermatol Online J. (2006) 12:7. doi: 10.5070/D339N411B8, PMID: 17083862

[3] Lucas EW Branton P Mecklenburg FE Moawad GN . Ectopic breast fibroadenoma of the vulva. Obstetrics Gynecology. (2009) 114:460–2. doi: 10.1097/AOG.0b013e3181af672d, PMID: 19622961

[4] Baisre A Heller DS Lee J Zheng P . Fibroadenoma of the vulva. A report of two cases. J Reprod Med. (2002) 47:949–51. doi: 10.1023/A:1020920104574, PMID: 12497689

[5] Dordević M Jovanović B Mitrović S Dordević G . Ectopic mammary tissue in vulva. Vojnosanitetski pregled. (2008) 65:407–9. doi: 10.2298/vsp0805407d, PMID: 18630137

[6] van der Putte SC . Mammary-like glands of the vulva and their disorders. Int J gynecological pathology: Off J Int Soc Gynecological Pathologists. (1994) 13:150–60. doi: 10.1097/00004347-199404000-00009, PMID: 8005737

[7] Bostwick DG Hossain D Qian J Neumann RM Yang P Young RH . Phyllodes tumor of the prostate: long-term followup study of 23 cases. J Urol. (2004) 172:894–9. doi: 10.1097/01.ju.0000134580.71261.57, PMID: 15310992

[8] Baset Z Arafat Y Amini J Rezaie F Mohammady N Mousavi SH . Intra-abdominal ectopic breast tissue in male patient presenting as a fibroadenoma: A case report. Ann Med Surg (2012). (2023) 85:1088–91. doi: 10.1097/ms9.0000000000000332, PMID: 37113935 PMC10129099

[9] Kim JH . Concurrent invasive carcinoma and fibroadenoma arising from bilateral ectopic breast tissue in the chest wall: A case report and literature review. J Korean Soc Radiol. (2024) 85:813–9. doi: 10.3348/jksr.2023.0137, PMID: 39130797 PMC11310427

[10] AlHarmi RAR Alawi N Al-Hashimi F Almehza J . Fibroadenoma in a suprapubic accessory breast. BMJ Case Rep. (2021) 14:e242665. doi: 10.1136/bcr-2021-242665, PMID: 34497052 PMC8438735

[11] Hilts A Suri R Machan M Singh U . Cutaneous periumbilical fibroadenomas: A rare case of ectopic breast tissue. Cureus. (2021) 13:e17523. doi: 10.7759/cureus.17523, PMID: 34603893 PMC8475742

[12] Ojo AB Sonusi SE Ayoade BA Oyedele AB . Unusual presentation of fibroadenoma in the pelvic region - a case report. Int J Surg Case Rep. (2024) 124:110352. doi: 10.1016/j.ijscr.2024.110352, PMID: 39341162 PMC11467574

[13] Robichaud S Tran-Thanh D Zhang ZP Durocher F Rahimi K . Lactating vulvar adenoma associated with fibroadenoma. Int J Surg Pathol. (2025) 33:1473–6. doi: 10.1177/10668969251314125, PMID: 39962834 PMC12276389

[14] Denlinger LN Lokhandwala PM Abendroth CS . Benign phyllodes tumor of the vulva: A case report and literature review. Rare tumors. (2015) 7:6010. doi: 10.4081/rt.2015.6010, PMID: 26788277 PMC4703923

